# Ordered Mesoporous Carbons with Well-Dispersed Nickel or Platinum Nanoparticles for Room Temperature Hydrogen Adsorption

**DOI:** 10.3390/molecules28186551

**Published:** 2023-09-10

**Authors:** Barbara Szczęśniak, Sylwia Głowniak, Jakub Woźniak, Stanisław Popiel, Jerzy Choma, Mietek Jaroniec

**Affiliations:** 1 Faculty of New Technologies and Chemistry, Military University of Technology, 00-098 Warsaw, Poland; sylwia.glowniak@wat.edu.pl (S.G.); jakub.wozniak01@wat.edu.pl (J.W.); stanislaw.popiel@wat.edu.pl (S.P.); jerzy.choma@wat.edu.pl (J.C.); 2 Department of Chemistry and Biochemistry & Advanced Materials and Liquid Crystal Institute, Kent State University, Kent, OH 44242, USA; jaroniec@kent.edu

**Keywords:** ordered mesoporous carbons, ball milling, metal decoration, hydrogen adsorption

## Abstract

A facile mechanochemical method was used for the synthesis of ordered mesoporous carbons (OMCs) with well-dispersed metal nanoparticles. The one-pot ball milling of tannins with a metal salt in the presence of a block copolymer followed by thermal treatment led to Ni- or Pt-embedded OMCs with high specific surface areas (up to 600 m^2^·g^−1^) and large pore volumes (up to ~0.5 cm^3^·g^−1^). The as-prepared OMC-based samples exhibited hexagonally ordered cylindrical mesopores with narrow pore size distributions (average pore size ~7 nm), which implies sufficient long-range copolymer-assisted self-assembly of the tannin-derived polymer upon milling even in the presence of a metal salt. The homogenous decoration of carbons with small-sized metal (Ni or Pt) particles was essential to provide H_2_ storage capacities up to 0.33 wt.% at 25 °C and under 100 bar. The presented synthesis strategy seems to have great potential in the practical uses of functionalized polymers and carbons for applications in adsorption and catalysis.

## 1. Introduction

Saccharides with low molecular weight and biomass-derived phenolic monomers are sustainable precursors for the synthesis of ordered mesoporous carbons (OMCs) [1,2]. Initially, OMCs were synthesized through a time-consuming procedure known as evaporation-induced soft-assembly (EISA) using block copolymers as soft templates. In this method, aldehydes such as formaldehyde or glyoxal were added as crosslinking agents to induce the copolymer-assisted assembly of carbon precursors into supramolecular mesostructures [3,4]. Later, researchers evidenced that some carbon precursors, e.g., tannins, can undergo self-condensation polymerization reaction without the use of any cross-linking agents [3,4,5]. Especially useful in the synthesis are plant-derived polyphenols such as hydrolyzable tannins (e.g., gallic, digallic, and ellagic acids) or condensed tannins (e.g., mimosa tannins). For instance, tannins extracted from mimosa trees with a molecular weight ranging from 500 to 3500 are inexpensive, renewable, and commercially available sources of natural polyphenols; world production is about 220,000 tons per year [6]. These polyphenols can self-condense without additional cross-linkers, forming three-dimensional interconnected networks through covalent bonds [7,8,9]. The pyrogallol- and catechol-like sites in tannins provide hydrogen bonds to hydrophilic blocks of the copolymers, which initiate the coordination polymerization reaction (crosslinking). Interestingly, the reaction can be performed within a short time, even in solid-state, e.g., using a high-speed ball milling process [6,7]. Mechanochemically-induced crosslinking of tannins complies well with the concepts of green chemistry. Castro-Gutiérrez et al. [7] synthesized mesostructured tannin-derived polymers in the presence of block copolymers and small water fraction via a high-speed ball milling of the extracts yielding OMCs after carbonization at 900 °C for 3 h. The procedure afforded well-ordered porous carbons with a specific surface area (SSA) of 588 m^2^·g^−1^ and mesopore volume (V_meso_) of 0.34 cm^3^·g^−1^. It was demonstrated that a similar synthesis process is also applicable for the preparation of nickel-decorated OMCs, just by adding nickel(II) acetate to the milling system [6]. In brief, mechanochemical mixing of mimosa tannin, triblock copolymers, and a nickel salt, followed by carbonization at 450 °C afforded Ni-decorated OMCs with high SSA of 1000 m^2^·g^−1^ and hexagonally ordered mesopores of about 7 nm. The as-obtained carbons possessed high content (up to 16 wt.%) of well-dispersed and small-sized (i.e., 5.4 nm) Ni nanoparticles. The authors claimed that the high dispersion of metal particles was possible due to the constraint of the mesoporous carbon support, which assured their separation and hence limited their aggregation at high temperatures (up to 600 °C). The as-obtained Ni-embedded OMCs showed promising activity in the hydrogenation process of bulky molecules. Similar milling-assisted strategies involving tannins as a main carbon source were also used to synthesize, e.g., alkaline metal oxide/mesoporous carbon composites [10], ruthenium cluster-embedded OMCs [11] or cobalt-embedded OMCs [12]. There is a great interest in exploring mechanochemical procedures for the preparation of functionalized carbon-based porous materials because this approach offers a fast and facile synthetic route that can completely meet the requirements of green chemistry [13].

It is still a challenge to develop porous sorbents capable of storing enough hydrogen under acceptable conditions (temperature and pressure) to accomplish the targets required for onboard applications [14,15]. In this field, the most promising classes of sorbents are carbon-based nanoporous materials. However, the hydrogen adsorption capacity of pure carbons, e.g., activated carbons under ambient temperature, is, to date, well below the onboard targets. For instance, their H_2_ adsorption capacity is up to 1–3 wt.% under very high pressure (i.e., 500 bar) [14,16]. The low uptake of molecular hydrogen at room temperature is limited by the low isosteric heat of hydrogen adsorption on activated carbons, which is usually in the range of 5–9 kJ mol^−1^ [14,17]. Optimization of structural parameters (porosity, pore size, and pore volume) can improve the hydrogen storage capacity to some extent, but it seems that a real breakthrough is only possible via embedding metals capable of causing spillover effect (e.g., Pt, Pd, Ni) [18,19]. The strategy is very promising to increase the isosteric heat of hydrogen adsorption and thereby enhance the strength of hydrogen interaction with the sorbent surface. The specially designed metal-decorated carbons have become one of the most preferred and promising nanosized materials in hydrogen storage technology; thus, they deserve further studies, also toward the development of more environmentally friendly and easy-to-scale-up synthesis procedures.

In this study, a facile mechanochemical recipe was used for the synthesis of carbons, including OMCs with well-dispersed Pt or Ni nanoparticles for hydrogen adsorption. For instance, the presented synthesis afforded small-sized Pt nanoparticles with a predominant size below 5 nm in the resulting carbons, no matter whether a soft template was used or not. In fact, the presence of the Pluronic template during the mixing of all reactants and the milling-induced polymerization of tannin, had a rather negative effect on the size of the embedded nanoparticles in the derived carbons. Furthermore, a thorough analysis was performed to present a reliable characterization of the synthesized samples, including the attempt to answer the question: What is the role of the porous structure as well as the presence of metallic nanoparticles (spillover effect) in hydrogen adsorption? It was also demonstrated that the ball milling-assisted synthesis procedure seems to be quite universal and available for use in the preparation of diverse metal-decorated polymers and carbons, which is of significant practical interest. Moreover, it is shown that the mechanochemical synthesis used permits fine-tuning of the composition and porosity of the resulting metal-decorated sorbents and catalysts.

Although this work shows some advantages of using such OMC materials with highly dispersed Pt nanoparticles for H_2_ adsorption, their other adsorption and catalytic applications can be enhanced by the uniformity (not necessarily ordering) of mesopores and the high dispersion of Pt nanoparticles.

## 2. Results and Discussion

**Figure 1:**
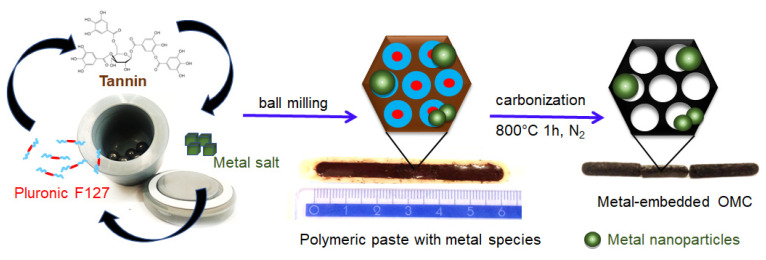
Schematic illustration of the mechanochemical synthesis of ordered mesoporous carbons decorated with metal nanoparticles, including digital images of the as-obtained dense polymeric paste after milling and carbon-based solid pieces after carbonization.

Natural plant polyphenols are far superior and sustainable candidates for the synthesis of mesoporous carbons than resorcinol because they have similar properties but are more environmentally friendly. Plant polyphenols such as tannins are rich in catechol or galloyl groups, which can be easily and strongly chelated with metal ions through coordination bonds. In this study, the plant tannin extracted from the mimosa tree was used as a carbon source, and Pluronic triblock copolymer PEO_98_-PPO_67_-PEO_98_ was used as a pore-forming agent. Firstly, micelles of amphiphilic block copolymer were generated under milling conditions in the presence of a small amount of water in a planetary ball miller. Next, Ni or Pt salt was added to the mixture, and milling was continued within 10 min to uniformly disperse it in the system. Afterward, tannin powder was added, and all reactants were milled together. The catechol and pyrogallol groups from tannin interacted with the hydrophilic corona of the block copolymer —PEO segments via hydrogen bonds, forming a continuous network structure composed of the self-organized polymer/polymer mesophase. The present metal species in the reaction system further strengthen the crosslinking of tannins around the template micelles through additional coordination bonds generated with the phenolic hydroxyl groups of tannins. The obtained gel-like composites were carbonized in a nitrogen atmosphere to decompose the soft template and obtain the ordered mesoporous carbon materials decorated with metal species. During heating, metal salts were first transformed into oxides and then reduced to metal nanoparticles at higher temperatures. Importantly, the coordination polymer used allows for the in situ reduction of metal salts into metal nanoparticles, which remain confined in a highly dispersed state in the carbon matrixes. A schematic illustration of the synthesis of the metal-decorated OMCs based on the ball milling-induced self-assembly process is presented in Figure 1. The nickel-decorated OMCs were labeled as OMCNix, and the platinum-decorated OMCs as OMCPtx, where x refers to the assumed wt.% of metal content. Two reference samples synthesized with the corresponding metal salt but without the addition of a Pluronic template were denoted as CNi3.5 and CPt3, respectively.

### 2.1. Morphological and Structural Characterization

**Figure 2:**
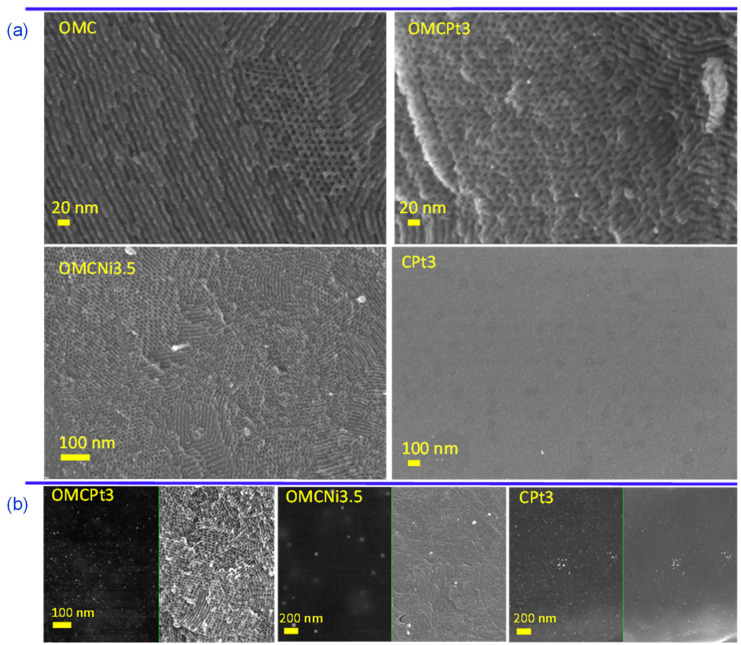
(**a**) SEM images of the selected samples, (**b**) additional SEM images (right side) and their counterparts (left side) showing the good dispersion of metal nanoparticles visible as white spots, obtained using the angle selective backscattered (AsB) detector.

**Figure 3:**
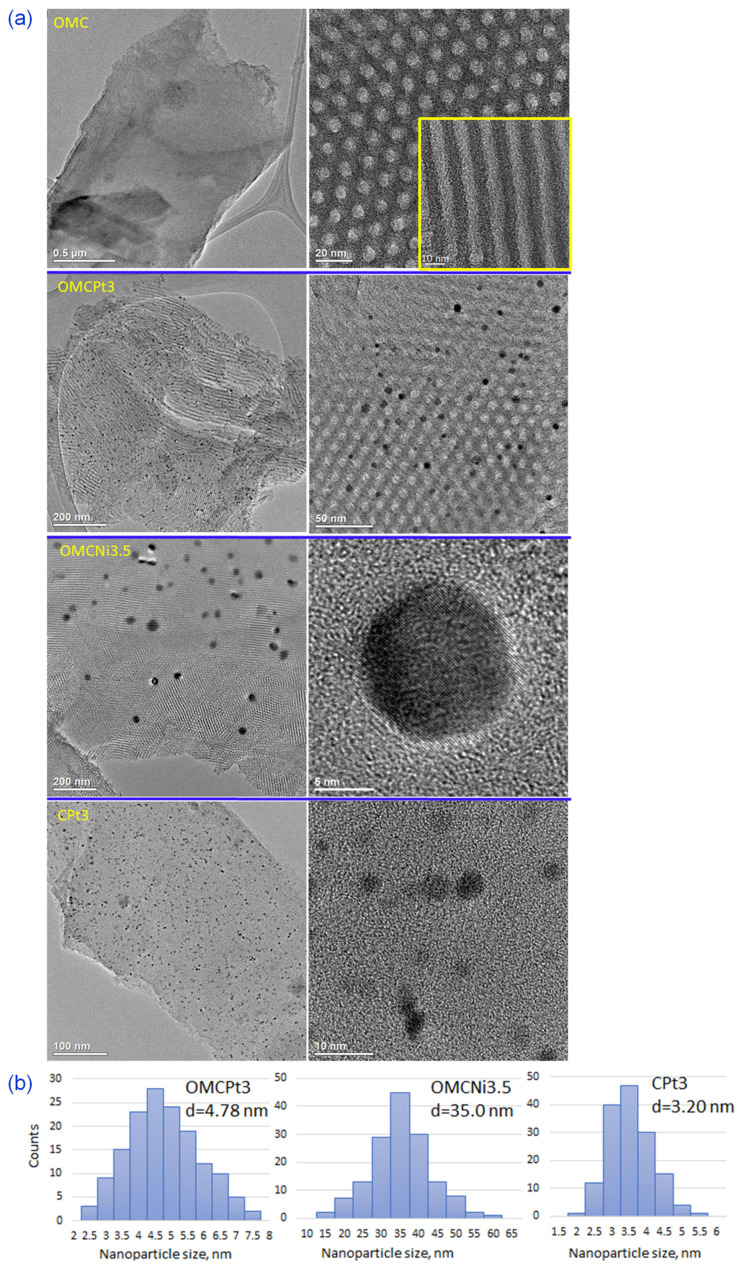
(**a**) TEM images of the selected samples, and (**b**) the corresponding nanoparticle size distributions calculated based on about 150 particles randomly selected particles, d—average nanoparticle size.

The uniform two-dimensional (2D) hexagonal arrays of mesopores are clearly visible already on SEM images (Figure 2a). Interestingly, the synthesis method, i.e., using high-speed ball milling, afforded carbons composed of relatively small randomly oriented domains of ordered uniform mesoporous channels, which can be beneficial from the viewpoint of adsorption kinetics, easier accessibility of micropores, and catalysis-related applications. Although SEM and TEM imaging techniques provide evidence about structural ordering at the local level, the measured N_2_ adsorption isotherms (see later the analysis of adsorption data) show steep capillary condensations steps, confirming that the observed ordering on the images shown in Figure 2 indicate that these mesoporous carbon samples are entirely ordered. The steep capillary condensation steps on these isotherms indicate the presence of uniform mesopores in the entire carbon samples, which, in combination with SEM images, implies that the mesopores present in these samples are entirely ordered. SEM images of metal-embedded OMCs indicate the undistorted copolymer-assisted assembly process in the presence of a metal salt, whereas the sample obtained without the addition of a Pluronic template (CPt3) exhibits rather smooth surface without visible nanopores (meso- and macropores), which is consistent with adsorption data. Figure 2b presents images of the selected Pt- or Ni-decorated samples, in which the right side shows SEM images and the left side shows the same images but acquired using the angle selective backscattered (AsB) detector. The visible light spots on these images with dark backgrounds (on the left) indicate the presence of small, well-dispersed metal nanoparticles in all selected samples. The metal nanoparticles are clearly visible in the TEM images and are quite evenly distributed in the synthesized carbons (Figure 3a). The presented TEM images, as well as the calculated corresponding nanoparticle size distributions, imply that Pt-decorated samples possess smaller nanoparticles, with an average size of 3–5 nm, compared to the exemplary Ni-decorated sample, which exhibited relatively large nanoparticles with an average size of 35 nm (Figure 3b). Interestingly, nanoparticles with an average size as small as 3.20 nm were observed in the CPt3 sample, obtained without the addition of template micelles, which shows that the ball milling process is highly efficient in merging metal salts with polymerizable carbon precursors yielding homogeneously distributed small-sized nanoparticles embedded in the carbon matrix. As mentioned above, tannins contain abundant pyrogallol and catechol functional groups, which serve as chelating sites and hence provide suitable coordination interactions between them and metal species. The analogous Pt-decorated sample, but containing ordered mesopores (OMCPt3), exhibits slightly larger Pt nanoparticles having an average size of 4.78 nm (Figure 3b). Apparently, large template micelles used in the synthesis may pose steric and/or adhesive obstacles, thus playing a rather negative role in the superior metal ions distribution within the tannin-derived polymer. The outcome does not support the previously reported results, implying the beneficial aspect of mesostructural support on both separation and limited aggregation of metal nanoparticles at high temperatures [6]. The ICP-MS analysis was conducted to provide the exact amounts of Ni and Pt, which were incorporated in the selected samples. The Ni content in sample OMCNi3.5 was 1.26 wt.%, and the Pt contents in samples OMCPt3 and CPt3 were 2.04 and 2.68 wt.%, respectively. Using the same amounts of tannin and platinum salt during the synthesis of both Pt-containing samples, the smaller amount of metallic Pt was in OMCPt3, which can be partially attributed to its open mesoporous structure and the corresponding higher losses of Pt species upon template decomposition, while the constricted microporous structure of CPt3 may trap more Pt.

**Figure 4:**
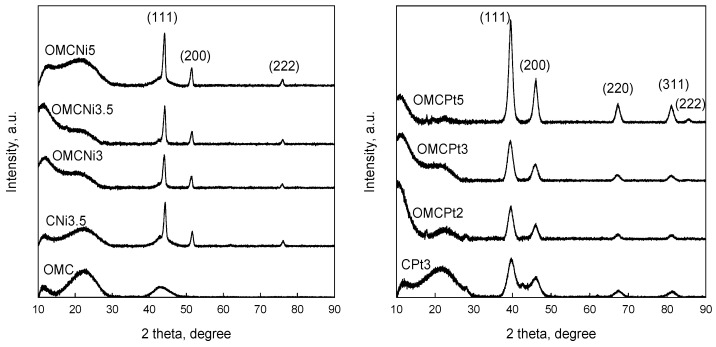
XRD patterns of all samples studied.

Powder XRD patterns of all samples studied are shown in Figure 4. The appearance of diffraction peaks at 2θ 44.1°, 51.5°, and 76.1° in the XRD patterns of Ni-containing samples indicates the presence of metallic Ni in the carbons. The formation of metallic Ni nanoparticles can be attributed to the reduction of NiO by neighboring carbon atoms, which can take place at even 450 °C under a nitrogen atmosphere, as reported earlier [6]. Pt-containing samples display the characteristic diffraction peaks at 39.5°, 46.1°, 67.3°, 81.2°, and 85.5° corresponding to (111), (200), (220), (311), and (222) planes of metallic Pt. These peaks are clearly wider than the characteristic peaks in the patterns of Ni-containing carbons, suggesting differences in the size of the embedded nanoparticles depending on the metal. The average crystalline sizes calculated by Scherrer’s equation were 5.5, 15.0, and 4.7 nm for OMCPt3, OMCNi3.5, and CPt3 samples, respectively. The presence of small-sized Pt nanoparticles in the Pt-containing samples is confirmed by the XRD analysis. However, the estimated values differ from those obtained from TEM images, especially for the Ni-containing OMCs. According to the XRD data, Ni nanoparticles in the carbons studied exhibit, on average, a smaller size of about 15 nm.

**Figure 5:**
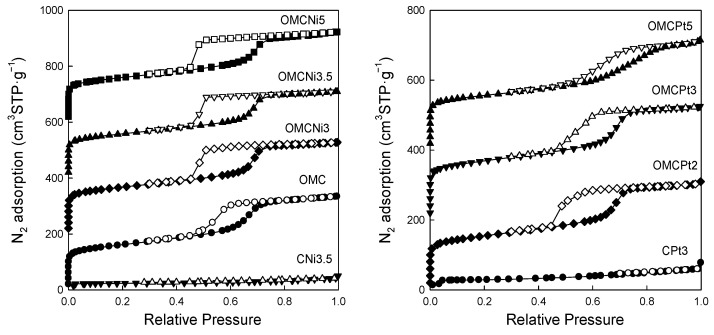
Nitrogen adsorption (closed symbols) and desorption (open symbols) isotherms measured at −196 °C for all samples studied.

**Figure 6:**
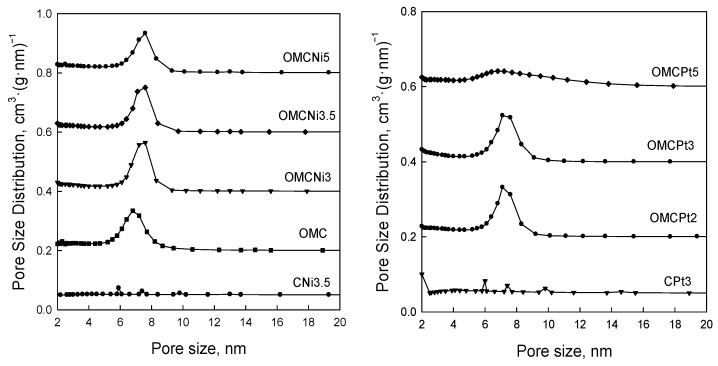
Pore size distribution functions determined by using the KJS method for all samples studied.

**Figure 7:**
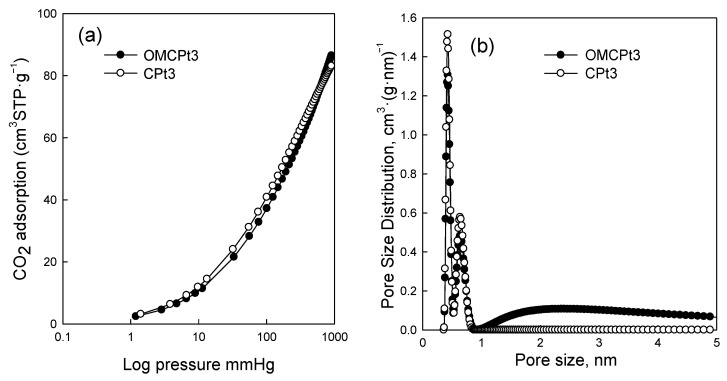
(**a**) CO_2_ adsorption isotherms at 0 °C, and (**b**) the corresponding pore size distribution functions determined by using the DFT method for OMCPt3 and CPt3.

Figure 5 shows nitrogen adsorption-desorption isotherms measured at −196 °C for all samples studied, and Figure 6 presents the corresponding pore size distributions (PSD) curves determined using the Kruk–Jaroniec–Sayari (KJS) method. Some adsorption-desorption isotherms in Figure 5 were shifted vertically by 200 cm^3^ STP·g^−1^ for clarity. Isotherms measured for all samples studied, except those synthesized without the addition of a Pluronic template (CNi3.5 and CPt3), are type IV according to the IUPAC classification characteristic for mesoporous materials [20]. The most efficient self-assembly between tannin and an amphiphilic copolymer occurs in an economically friendly environment of a small amount of pure water. The addition of water also had a beneficial impact on the form of the resulting paste after milling, and the used water to tannin ratio afforded a uniform tight paste that easily detaches from milling tools, which is crucial from the practical viewpoint. The OMCs decorated with Ni and Pt nanoparticles feature high both SSAs up to 600 m^2^·g^−1^ and V_t_ up to 0.51 cm^3^·g^−1^, comparable to those of pure OMC sample, and uniform pores (except OMCPt5 sample), indicating undistorted assembly of Pluronic copolymer and tannin in the presence of metal salt during high energy milling process. An average mesopore size in these samples is around 7 nm, and only OMCPt5 with the largest amount of Pt content shows broad PSD function, identifying its nonuniform mesostructure. The samples CNi3.5 and CPt3, which were synthesized without the addition of a mesopore-directing agent, show extremely reduced porosity according to nitrogen adsorption data; their SSAs are up to 100 m^2^·g^−1^, V_t_ up 0.12 cm^3^·g^−1^, thus implying the essential role of the polymeric template on the porosity in the synthesized products, especially mesoporosity, see Table 1. However, one wonders why the micropore volumes are only up to 0.04 cm^3^·g^−1^ for the CNi3.5 and CPt3, therefore about four times lower than the values determined for OMC-based samples. Microporosity is generated during the carbonization of tannin, and thus, the narrowest porosity should be similar for all carbons studied, no matter if the mesopore-directing agent was used or not. Moreover, micropores are clearly visible on the TEM image of CPt3, see Figure 3a. Therefore, carbon dioxide adsorption at 0 °C was measured for the corresponding samples CPt3 and OMCPt3 to further study the presence of small micropores in their structure. As expected, the examined tannin-derived carbons possess similar PSD functions in the range of narrow microporosity, and the calculated pore volumes up to 0.8 nm were about 0.20 cm^3^·g^−1^ for both samples (Figure 7). The value of the specific surface area determined for CPt3 from the CO_2_ adsorption data by using density functional theory (DFT) was 837 m^2^·g^−1^, whereas the OMCPt3 sample exhibited a DFT surface area of 1060 m^2^·g^−1^. Thus, including the detected narrow microporosity, the difference in the surface areas is not as considerable as it was determined from N_2_ adsorption data. Apparently, CO_2_ adsorption isotherms are well suited to determine the micropore volume for carbons with narrow microporosity, such as tannin-derived carbons, because CO_2_ at 0 °C has a high saturation pressure and thus allows the study of the narrowest porosity. Contrarily, the low temperature and the low relative pressure at the beginning of the N_2_ adsorption measurements cause diffusional limitations; namely, N_2_ molecules cannot reach the narrowest micropores [21].

### 2.2. Hydrogen Adsorption

**Figure 8:**
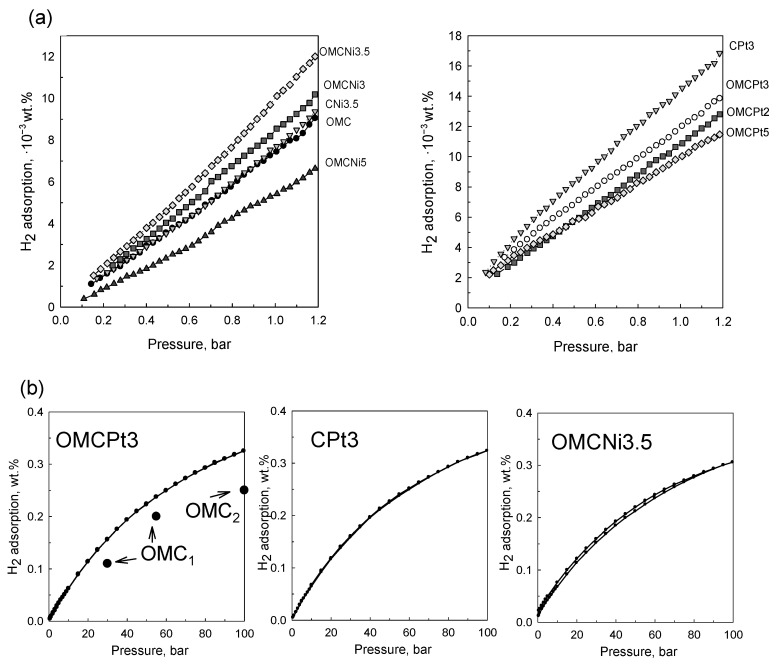
Hydrogen adsorption isotherms determined at 25 °C (**a**) for all samples studied under pressure up to 1.2 bar, and (**b**) for the selected samples under pressure up to 100 bar. The figure contains literature data for bare OMC_1_ [24] and OMC_2_ [23] samples for comparison.

The decoration of OMC with small amounts of Ni and Pt (~1.3–2.0 wt.%) resulted in enhanced H_2_ adsorption capacities, already under 1 bar at 25 °C, due to the spillover effect. Hydrogen spillover is a surface phenomenon relying on the dissociation of hydrogen molecules on metal sites, followed by migration and binding of the resulting hydrogen atoms to carbon layers [18]. Among OMC-based samples, the highest hydrogen uptake at 1 bar of 12.0 × 10^−3^ wt.% was obtained for OMCPt3 containing 2.04 wt.% of metallic Pt. The sample containing 1.26 wt.% of metallic Ni (OMCNi3.5) adsorbed the highest amount of H_2_ (10.2 × 10^−3^ wt.%) among Ni-doped OMCs and, in comparison, to the bare OMC, which had an H_2_ adsorption capacity of 7.4 × 10^−3^ wt.% at the same conditions (Figure 8a). The sample containing 2.68 wt.% of the smallest Pt nanoparticles prepared without the addition of template (CPt3) showed the highest H_2_ adsorption capacity under 1 bar of 14.6 × 10^−3^ wt.%, probably due to the optimal amount of the embedded small-sized metal nanoparticles among all samples studied in addition to the similar volume of fine micropores in all samples (see discussion below). It seems that the physicochemical nature of hydrogen adsorption on the metal-containing sorbent surfaces supports the existence of a spillover effect. To further understand the adsorption phenomenon and specifically compare the narrow microporosity in the samples studied, the pore size distributions were additionally determined from CO_2_ adsorption data. The examined tannin-derived carbons possess similar microporosity up to 0.8 nm, and thus, the role of the two factors, porosity and nanoparticles, for the low-pressure H_2_ adsorption can be assessed according to the results of this study. Apparently, having the optimal metal decoration (type, quantity, dispersion, size of nanoparticles) on the surface of porous carbons, the effect of metal nanoparticles on the overall hydrogen adsorption is roughly up to 50%. Thus, the best-decorated sample adsorbed 14.6 × 10^−3^ wt.% of hydrogen, whereas the adsorption of the bare carbon was about 7.4 × 10^−3^ wt.%, and as mentioned, all samples have similar narrow porosity and yet the smallest pores contribute the most to the low-pressure H_2_ uptake. Note that the adsorption of H_2_ molecules on porous carbons is physical in nature and mainly driven by high specific surface area, which is significantly enlarged by fine micropores and not by the well-developed mesoporosity. However, the presence of mesopores improves adsorption kinetics that is evidenced by unmeasurable N_2_ adsorption on non-mesoporous CPt3 carbon sample at −196 °C (compare the micropore volumes from N_2_ and CO_2_ adsorption data for this sample provided in Table 1). In addition, it is known that hydrogen adsorption/desorption on carbons is fully reversible due to the very small size of H_2_ and the physical nature of adsorption [22]. Finally, the comparable enlargement of H_2_ adsorption on the metal-decorated carbons and similar microporosity in all samples indicate that most metal nanoparticles are accessible to very small H_2_ molecules.

Under high hydrogen pressure of around 100 bar, the selected samples decorated with Pt or Ni nanoparticles showed very similar adsorption capacities. The highest adsorption was observed for Pt-containing carbons: OMCPt3 adsorbed 0.33 wt.%, and CPt3–0.32 wt.%, whereas OMCNi3.5 adsorbed as high as 0.30 wt.% of hydrogen at 25 °C (Figure 8b). Hydrogen adsorption/desorption hysteresis is not observed for both Pt-decorated carbons, suggesting that the adsorbed hydrogen can be fully desorbed and, thus, their surface recovered for another adsorption/desorption cycle, which is highly desirable from the viewpoint of hydrogen storage applications. In the case of the Ni-containing OMC, the desorption isotherm is slightly above the adsorption curve, which implies that a very small amount of hydrogen species was not spilled over to the carbon support and could remain on the metal surface [23]. This phenomenon may be attributed to larger Ni nanoparticles than those in the Pt-containing samples. Hydrogen adsorption depends on the specific surface area (porosity) of the carbon support as well as the dispersion and size of metal nanoparticles; e.g., reducing the size of metallic clusters can significantly increase metal-carbon contact, which is beneficial for the spillover effect. However, the high storage efficiency of Ni-decorated OMC implies that nickel is a good candidate to substitute precious metals such as Pt or Pd, usually proposed as active sites in carbonaceous sorbents for high hydrogen adsorption. For comparison, hydrogen storage capacity obtained for pure OMC sample at 25 °C is 0.2 wt.% under 55 bar [24] and around 0.25 wt.% under 100 bar [23] (Figure 8b). Apparently, the enhancement in the H_2_ adsorption caused by the spillover effect would be enlarged under higher gas pressures [23]. Overall, the high hydrogen adsorption performance of metal-decorated carbons was mostly attributed to the small-sized, confined, and hence highly dispersed metal nanoparticles in the carbon matrixes rather than to the mesoporous features of the carbon support. However, mesoporosity may have some influence, especially under higher gas pressures, which is associated with the facilitated gas transfer and easier accessibility of the metallic active sites for hydrogen molecules.

Hydrogen adsorption capacities of diverse carbon-based materials at 25 °C and different pressures are shown in Table 2 for comparison. The metal-decorated OMCs synthesized in this work exhibited comparable or lower H_2_ adsorption capacity compared to other carbons presented in the literature. However, one should also note that the usually reported hydrogen adsorbents are prepared via tedious synthesis procedures and/or using no sustainable carbon precursors/reagents. The differences in the hydrogen adsorption capacities result from dissimilar surface area and porosity, metal content, nanoparticle size, and dispersion in the adsorbent structure, among others. Moreover, the comparison is often not straightforward due to differences in experimental conditions applied for hydrogen adsorption measurements.

## 3. Materials and Methods

### 3.1. Chemicals

All chemicals were analytically pure and used as received without further purification. Mimosa tannin extract was kindly provided by the company SilvaChimica (Italy). Poly (ethylene oxide)–poly (propylene oxide)–poly (ethylene oxide) triblock copolymer Pluronic F127, nickel(II) nitrate hexahydrate, and potassium tetrachloroplatinate(II) were supplied by Merck. Note that Pluronic polymers are nontoxic, inexpensive, and commercially available, while tannin is a biomass-type carbon precursor, which makes the synthesis more sustainable and greener.

### 3.2. Mechanochemical Synthesis of Metal-Decorated Ordered Mesoporous Carbons

A pure OMC reference sample was prepared using a strategy reported by our team previously [29], which was a modified procedure adopted from the literature [7]. OMCs with well-dispersed metal nanoparticles were synthesized by adding metal salts at the right moment during the synthesis of OMC. Briefly, 1 g of Pluronic F127 and 2 mL of distilled water were initially milled for 5 min with a rotation speed of 500 rpm in a Planetary Mono Mill PULVERISETTE 6 classic line, ball milling machine (Fritsch, Bahnhofstraße, Germany). The device was equipped with a 45 mL ceramic milling bowl and 8 stainless, ceramic balls of diameter 1 cm. Next, a certain amount of metal salt was added, and milling was maintained for an additional 10 min. Afterward, 2 g of mimosa tannin was added, and the mixing was continued for 1 h. A uniform and easy-to-handle paste-like material (easily detaches from bowl and balls just in one tight piece) was directly transferred to a quartz boat and carbonized directly at 800 °C for 1 h under flowing nitrogen, a heating rate was 2 °C·min^−1^. The as-obtained sample was cooled down naturally under flowing nitrogen and was then immersed in a small amount of ethanol-distilled water (1:1) solution; afterward, it was rinsed with water and dried at 80 °C.

### 3.3. Measurements and Calculations

X-ray diffraction analysis (XRD), nitrogen, and low-pressure hydrogen adsorption isotherms were used to determine crystallographic phases, porous structure, and H_2_ adsorption capacities under 1 bar of all samples, respectively. The XRD analysis was conducted using the Bruker D2 PHASER diffractometer with Cu Kα X rays operating at 30 kV and 10 mA, in the range of 10° < 2*θ* < 90° with a step of 0.015° and acquisition time of 2 s per step at room temperature. Nitrogen and low-pressure H_2_ adsorption isotherms were measured at −196 °C and 25 °C, respectively, using the ASAP 2020 volumetric analyzer manufactured by Micromeritics Instrument Corp. Experimental errors associated with the measured adsorption data using commercial adsorption analyzers have been discussed elsewhere [30]. All samples were outgassed at 300 °C for 5 h before adsorption measurements. The specific surface area was estimated using the Brunauer-Emmett-Teller (BET) method based on low-temperature nitrogen adsorption isotherm data in a relative pressure (p⋅p_0_^−1^) range of 0.05–0.25 [31]. The total pore volume (V_t_) was calculated using the volume of the nitrogen adsorbed at a relative pressure of ≈0.99. The pore size distributions (PSDs) were determined by using the BJH (Barrett–Joyner–Halenda) algorithm with the Kruk–Jaroniec–Sayari (KJS) correction for adsorption branches of the nitrogen isotherms by applying the thickness curve based on Harkins and Jura equation [32]. For some samples, CO_2_ adsorption isotherms were determined at 0 °C using the ASAP 2020 analyzer, and the corresponding PSD functions, values of the surface areas, and micropore volumes up to 0.8 nm were calculated by using the density functional theory (DFT), for the purpose of comparison.

Additionally, scanning electron microscopy (SEM), transmission electron microscopy (TEM), high-pressure hydrogen adsorption, and inductively coupled plasma mass spectrometry (ICP-MS) were performed for the selected samples. SEM images were obtained using the scanning electron microscope LEO 1530 manufactured by Zeiss operated at 2 kV acceleration voltage. TEM images were taken on a FEI Titan G2 60-300 microscope operated at 300 kV acceleration voltage. Hydrogen adsorption-desorption isotherms up to 100 bar were measured using an iSorb HP1 100 high-pressure gas sorption analyzer manufactured by Anton Paar Quantatec. The content of nickel and platinum in the selected samples was measured by ICP-MS (iCAP RQ ICP-MS, Thermo Scientific, Bremen, Germany). The instrument was equipped with a MicroMist borosilicate nebulizer (Glass Expansion). The samples were first mineralized using a High-Performance Microwave Digestion System (ETHOS UP, Milestone).

## 4. Conclusions

The presented facile mechanochemical route can be used for the synthesis of ordered mesoporous carbons decorated with small metallic nanoparticles from sustainable biomass-derived precursor—tannin extracts. These results indicate the successful cooperative block copolymer-assisted self-assembly of tannins in the presence of metal salts, leading to metal-decorated carbons with ordered and uniform mesopores after annealing at 800 °C. OMC-based samples exhibited specific morphology, i.e., small randomly oriented domains of ordered uniform mesopores. The ball milling-assisted synthesis afforded well-distributed small-sized Pt nanoparticles with a predominant size below 5 nm in the resulting carbons, whereas Ni-decorated OMC possessed larger nanoparticles with an average size of 35 nm. The decoration of carbons with Pt and Ni had a beneficial impact on their hydrogen adsorption capacities, providing H_2_ adsorption up to 0.33 wt.% at 25 °C and under 100 bar. The profound analysis of the carbons studied leads us to the conclusion that the effect of platinum nanoparticles on the low-pressure hydrogen adsorption on the metal-decorated porous carbons can be up to 50%. Carbon dioxide adsorption data are well suited to determine the micropore volume for carbons with narrow microporosity.

Overall, the presented mechanochemical method seems to be sustainable, scalable, and suitable for the synthesis of diverse metal-decorated materials, which may have a high potential in, e.g., adsorption- and catalysis-related applications.

## Figures and Tables

**Table 1:** Structural parameters evaluated from N_2_ adsorption at −196 °C, CO_2_ adsorption at 0 °C, and H_2_ adsorption capacities at 25 °C and 1 bar for the samples studied.

Sorbent	S_BET_ m^2^·g^−1^	V_t_ cm^3^·g^−1^	V_micro_ cm^3^·g^−1^	V_meso_ cm^3^·g^−1^	w nm	Mesoporosity %	H_2_ Adsorption ·10^−3^ wt.%
OMC	571	0.52	0.15	0.37	6.8	71	7.4
OMCNi3	595	0.51	0.17	0.34	7.6	67	8.6
OMCNi3.5	553	0.48	0.15	0.33	7.6	69	10.2
OMCNi5	566	0.50	0.16	0.34	7.6	68	5.4
OMCPt2	550	0.48	0.16	0.32	7.1	67	10.8
OMCPt3	600 (1060 *)	0.50	0.17 (0.19 *)	0.30	7.1	64	12.0
OMCPt5	558	0.49	0.18	0.31	6.8	63	10.0
CNi3.5	80	0.08	0.04	0.04	-	50	7.8
CPt3	100 (837 *)	0.12	0.03 (0.21 *)	0.09	-	75	14.6

Notation: SSA—BET specific surface area; V_t_—total pore volume obtained from the amount adsorbed at p⋅p_o_^−1^ ≈ 0.99; V_micro_—volume of micropores (pores < 2 nm) calculated by V_t_-V_meso_; V_meso_—the volume of mesopores (pores size between 2 to 50 nm) obtained based on the KJS PSD functions; w—average mesopore size; Mesoporosity—the percentage of the volume of mesopores (V_meso_) to the total pore volume (V_t_); *—specific surface area and micropore volume up to 0.8 nm obtained from the CO_2_ adsorption data at 0 °C, calculated by using DFT.

**Table 2:** Comparison of hydrogen adsorption capacity of different carbon-based materials at 25 °C and different pressures.

Sample	Hydrogen Adsorption, wt.%	Measurement Conditions (Temperature °C/Pressure Bar)	Ref.
OMC	0.20	25/55	[24]
CMK-3	0.24	25/45	[25]
Carbon nitride	0.05	25/45	[25]
Activated carbon	0.60	25/120	[26]
Pd-decorated N-doped reduced graphene oxide	0.46	25/10	[27]
Co-embedded OMC	0.45	25/55	[24]
Ni-CMK-3	0.20	25/30	[28]
Ni-decorated OMC	0.75 *	25/100	[23]
Pt-decorated OMC	0.50 *	25/100	[23]
Pd-decorated OMC	0.40 *	25/100	[23]
Pt-decorated carbon	0.32	25/100	This work
Pt-decorated OMC	0.33	25/100	This work
Ni-decorated OMC	0.30	25/100	This work

Notation: *—an approximate value of hydrogen adsorption under 100 bar read from the corresponding figure from ref. [23] for comparison.

## Data Availability

The data are available upon request to the authors.
